# “Finding meaning in life after a cancer diagnosis”: A mixed methods study with Latinos with advanced cancer

**DOI:** 10.1017/S1478951525101387

**Published:** 2026-01-28

**Authors:** Rosario Costas-Muniz, Normarie Torres-Blasco, Stephanie Mariah Nuñez, Luciana Olivero Dos Santos, Oscar Galindo-Vázquez, William Breitbart, Eida Castro

**Affiliations:** 1 Department of Psychiatry & Behavioral Sciences, Memorial Sloan Kettering Cancer Center, New York, NY, USA; 2 Weill Cornell Medical College, Department of Psychiatry, New York, NY, USA; 3 Department of Psychology, Ponce Research Institute, Ponce Health Sciences University, Ponce, Puerto Rico; 4 School of Brain and Behavioral Sciences, Clinical Psychology Department, Ponce Research Institute, Ponce Health Sciences University, Ponce, Puerto Rico; 5 The City College of New York, City University of New York, New York, NY, USA; 6 Gracuate School of Psychology, The New School, New York, NY, USA; 7 Integrative Oncology Unit, Instituto Nacional de Cancerología, Mexico, Mexico; 8 School of Medicine, Psychiatry Department, Ponce Research Institute, Ponce Health Sciences, Ponce, Puerto Rico

**Keywords:** Latinos, healthcare disparities, minority health, meaning, counseling, advanced cancer

## Abstract

**Objective:**

To determine associations between spiritual well-being (faith and meaning dimensions) with emotional suffering (anxiety, depression, hopelessness, and quality of life) in Latinos with advanced cancer and examine themes of existential coping.

**Design:**

In a mixed-methods study, participants were recruited from cancer clinics in New York and Puerto Rico. Measures included the Functional Assessment of Chronic Illness Therapy – Spiritual Well-Being Scale, the Hospital Anxiety and Depression Scale, and the Beck Hopelessness Scale. A subset of participants completed in-depth semi-structured interviews exploring the roles of existential and religious factors in adjustment to cancer. Correlations were conducted, and the interviews were analyzed with a thematic analysis approach.

**Results:**

A sample of 142 Latinos with advanced cancer participated (67.6% stage IV and 32.4% stage III). The spiritual well-being, faith and meaning factor were associated with anxiety and depression symptoms. Meaning was associated with lower hopelessness and showed stronger associations with emotional suffering than the faith dimension. Lower acculturation was associated with higher hopelessness but not with depression/anxiety. In semi-structured interviews (*n* = 24), recurrent themes were: (1) receiving existential support from counselors; (2) receiving spiritual support from family and/or friends; (3) focusing on being spiritual and finding purpose rather than on a specific religion or faith; (4) religious coping; and (5) spiritual coping, focused on self-growth, finding meaning, and helping others to cope. Patients identified sources of meaning, including helping others, having a fighting spirit, a spirit of learning, enjoying work, enjoying life, family and children, confidence in providers/treatment, God/faith, and spirituality.

**Significance of results:**

Meaning had a more significant influence than faith on emotional suffering. Participants emphasized the importance of finding meaning and purpose, self-growth, and helping others as ways to cope with an advanced diagnosis. Interventions with a meaning-making approach, emphasizing finding purpose and growth, are needed for Latinos with advanced cancer.

## Introduction

Spirituality is considered an important dimension of quality of life in cancer (Peterman et al. 2002; Weaver and Flannelly 2004). It encompasses the beliefs and practices of religious communities, and it also includes dimensions of meaning-making, purpose, and transcendence in life (Oman 2013). One of the most widely used scales is the Functional Assessment of Chronic Illness Therapy: Spiritual Well-Being Scale (FACIT-Sp) (Canada et al. 2012; Peterman et al. 2002). The FACIT-Sp uses two factors to evaluate important aspects of spirituality: meaning/peace and faith. The meaning/peace factor assesses meaning, peace, and purpose in life; while the faith factor assesses the strength and effectiveness of one’s own faith (Canada et al. 2012; Peterman et al. 2002). Addressing spirituality in healthcare is essential for person-centered care, which is defined by the Institute of Medicine (2001) as “care that is respectful of and responsive to individual patient preferences, needs, and values and ensuring that patient values guide all clinical decisions.”

For Latinos, spirituality is an important core cultural value that can facilitate resilience in the face of cancer (Hunter-Hernandez et al. 2015). It can help Latino people with cancer to cope with active treatment and cope with the consequences of cancer during survivorship (Hunter-Hernandez et al. 2015). In a systematic review (Samuel et al. 2020) of the quality of life of Latino cancer survivors, spiritual well-being and spirituality-based coping were higher among Latino cancer survivors compared with other racial/ethnic groups in 50% of studies. These research articles also documented multiple religious and spiritual practices for coping with cancer, including prayer, faith, finding meaning and peace, religious or church attendance, and relationship with God (Samuel et al. 2020). The main findings were that Latino survivors commonly reported spirituality as a source of strength and comfort, and spirituality/religious coping was associated with lower anxiety, fatigue, and depression and higher general quality of life and functional well-being (Samuel et al. 2020).

In a study (Hunter-Hernandez et al. 2015) with a sample of Latina breast cancer survivors, spiritual well-being and the factors of meaning/peace and faith were associated with higher quality of life and lower levels of depression; and the meaning/peace factor was the main predictor of an increase in quality of life and a reduction in depression (Garduño-Ortega et al. 2021). Spiritual well-being and the meaning/peace subscales have also been found to correlate with lower financial toxicity in Latina breast cancer survivors (Echeverri-Herrera et al. 2022). Furthermore, in a longitudinal study with Latina women diagnosed with cancer, greater baseline spiritual well-being scores were associated with less concurrent distress, better quality of life, and emotional and functional well-being over time (Barata et al. 2022).

The association between spirituality and adjustment appears to be stronger for meaning- and peace-related well-being than for faith- or religious-related well-being in Latina survivors and Latinas undergoing cancer treatment (Barata et al. 2022; Garduño-Ortega et al. 2021). Nevertheless, religious concerns, practices, and coping are frequently present and sources of comfort among Latinos as they adapt to the cancer experience (Ashing-Giwa et al. 2006; Culver et al. 2004). Latinas diagnosed with breast cancer are more likely to seek and receive counseling from a spiritual leader, priest, or pastor after a cancer diagnosis (Costas-Muniz et al. 2021b, 2017). Additionally, mental health providers of Latinos with cancer cite religious and spiritual beliefs and concerns as ones that were addressed when counseling Latino patients (Costas-Muniz et al. 2021a).

However, few studies have focused on the existential needs of Latinos with cancer, and no studies have focused on the needs of Latinos with advanced cancer. Moadel et al. (1999) found that between 50% and 65% of Latinos with cancer reported wanting help to find hope, meaning in life, and spiritual resources, and wanting to talk to someone about finding peace and meaning in life. Among these patients, 25% to 51% reported unmet spiritual or existential needs, which included wanting help in overcoming fears, finding hope, talking about peace of mind, finding meaning in life and spiritual resources, and having someone to talk to about the meaning of life and death (Moadel et al. 1999).

Several systematic reviews (Teo et al. 2019; Uitterhoeve et al. 2004) examining interventions for people with advanced cancer, or at the end of life, have shown that the interventions with the most evidence-based findings are Meaning-Centered Psychotherapy (MCP) (Vos and Vitali 2018), dignity therapy (Li et al. 2020), and managing cancer and living meaningfully (Troncoso et al. 2019). Many of these therapies have been implemented in different modalities (e.g., individual, group), cancer types (e.g., breast), groups (e.g., caregivers, parents), settings (e.g., palliative care, hospice), and countries and populations (e.g., Chinese, Canadians) (Teo et al. 2019; Uitterhoeve et al. 2004). However, MCP is the only intervention adapted and tested for Latinos with advanced cancer (Costas-Muñiz et al. 2022; Costas-Muniz et al. 2020, 2023; Torres-Blasco et al. 2020a, 2020b; 2022b). Studies have shown that MCP is acceptable to Latinos from the USA (Costas-Muniz et al. 2020, 2023) and Puerto Rico (Torres-Blasco et al. 2020a, 2020b) and with Latino caregivers (Torres-Blasco et al. 2022a).

MCP is a manualized psychotherapy intervention developed for the specific spiritual and meaning-making needs of people with advanced disease (Breitbart 2002; Breitbart et al. 2018; Breitbart and Poppito 2014; Breitbart et al. 2010, 2012, 2015). The basic concepts of MCP include: (1) Meaning of life, (2) Desire to find meaning, and (3) Freedom to find meaning in existence and to choose the attitude toward suffering. The main sources of meaning in life are derived from creativity (work, deeds, and dedication to causes), experience (art, nature, humor, love, relationships, and roles), attitude (attitude one takes toward suffering and existential problems), and historical context (having a sense of past, present and future legacy is critical for meaning-making) (Breitbart 2002).

In this article, we examine associations between spiritual well-being, faith and peace/meaning, with quality of life, depression, anxiety, distress, and hopelessness. Given the prior evidence with samples of Latinos with cancer undergoing treatment and in remission, we hypothesize a strong association of peace/meaning with psychological adjustment in people with advanced disease. We also sought to quantitatively document the importance, acceptability, and relevance of existential themes as Latinos adjust to advanced cancer. Finally, we examined the narratives of meaning-making and sources of meaning for Latinos with advanced cancer and their alignment with the themes of MCP.

## Methods

### Study design and procedure

A mixed-methods, concurrent, integrative approach was used for this study. Quantitative and qualitative data were collected from Latinos with advanced cancer.

### Participants

Latino individuals with advanced cancer were recruited from two cancer clinics in New York City and one clinic in Ponce, Puerto Rico. Eligible patients had been diagnosed with advanced cancer (stages III or IV), identified as Latino, and were fluent in Spanish. After initial prescreening, which was limited to Latino/Hispanic ethnicity and Spanish surname documented in the medical record, 1274 people’s records were fully screened, and 267 people were eligible. Two hundred sixty-seven eligible people were approached by research staff from August 2015 through March 2022. Forty-six percent refused to participate (i.e., time constraints and lack of interest), yielding a sample of 142 people with advanced cancer. A nested sample of the first consecutive 24 participants was invited to complete in-depth, semi-structured interviews. All participants provided their written consent. This research was reviewed and approved by the institutional review boards (IRBs) of each institution: Memorial Sloan Kettering Cancer Center’s IRB #15-076, Lincoln Medical Center’s IRB#16-011, and Ponce Health Sciences University’s IRB #161129.

### Assessments

Both quantitative and qualitative assessments were used, which included validated scales, an investigator-developed survey, and a semi-structured interview guide. Demographic and medical information was abstracted from the patients’ medical records, including information regarding age, marital status, income, education, preferred language, place of residence, clinical diagnosis, clinical stage, treatment status, and type of treatments received (including cancer and psychiatric treatments). A brief demographic section was also added in the quantitative assessments that included questions about age, marital status, income, education, employment status, language, birth country, place of residence, clinical diagnosis, cancer stage, years since diagnosis, and treatments received. Medical records were used as the primary sources of information; data collected from the questionnaire were supplemental.

All the instruments included have been validated in Spanish. The FACIT Spiritual Well-Being Scale (FACIT-Sp-12) was used to assess the nature and extent of spiritual well-being (Peterman et al. 2002). This measure generates two sub-scales: Faith (the importance of faith/spirituality) and Meaning/Peace (one’s sense of meaning and purpose in life); scores range from 0 to 48 and it is validated for Spanish (Canada et al. 2012). The Spanish version (Herrero et al. 2003) of the Hospital Anxiety and Depression Scale (HADS) was used to measure clinical levels of depression and anxiety. The HADS consists of 14 items (score range 0–42), including a 7-item anxiety subscale (score range 0–21) and a 7-item depression subscale (score range 0–21).

The Spanish version (Aliaga 2006) of the Beck Hopelessness Scale (BHS) was used to measure degrees of pessimism and hopelessness.(Beck et al. 1974) The BHS includes 20 true/false questions (scores range 0–20). The Spanish version (Cella et al. 1998) of the Functional Assessment of Cancer Therapy – General (FACT-G) is a 27-item questionnaire composed of general questions divided into four main domains: physical well-being, social/family well-being, emotional well-being, and functional well-being (Cella et al. 1993). Finally, the main author and investigator developed a 27-item survey that included 22 questions assessing the acceptability of the goals and existential concepts and themes related to MCP, existential issues that would benefit from support, and important themes for counseling or psychotherapy, including spiritual and existential issues (Costas-Muniz et al. 2020, 2023).

The interview guide included open-ended questions about (1) patients’ meaning-making processes and coping, (2) sources of meaning in their lives, (3) spirituality, and (4) meaning-making after their cancer diagnosis. The exploration of sources of meaning was guided by the definition and use of these concepts in MCP:

1. Historical Sources of Meaning: life as a legacy that has been given (past), that one lives (present), and that one gives (future).
2. Attitudinal Sources of Meaning: encountering life’s limitations and choosing one’s attitude.
3. Creative Sources of Meaning: Engaging in life fully requires creativity, responsibility, courage, and accomplishments in life.
4. Experiential Sources of Meaning: Connecting with life, such as through love, beauty/nature, and joy/humor.

### Statistical analyses

Statistical analyses were performed using the IBM SPSS software, version 20 (IBM North America, New York, NY, USA). Descriptive statistics were utilized for sample characterization of demographic and clinical variables. Frequencies and percentages were reported for the items assessing the acceptability of the goals, existential concepts, and themes related to MCP. Pearson’s *r* correlations were used to evaluate associations between spiritual well-being factors, quality of life, and mood symptoms. Multivariate regression models were used to examine the association between spiritual well-being, meaning/peace, and faith with quality of life and mood symptoms. Models were adjusted for demographics (age, education, marital status, and birth country) covariates. A two-sided *p-*value of less than .05 was considered statistically significant.

#### Qualitative analyses

Qualitative analyses were guided by the seven steps of the Framework Method (Gale et al. 2013): (1) The 24 interviews were transcribed. (2) Three coders became familiar with the transcripts. (3) The coders used open coding (coding anything that might be relevant from as many different perspectives as possible) for the transcripts using the report and query functions of the qualitative analysis software, ATLAS.ti. (4) All coders met to discuss points of divergence and convergence. These discussions continued until the group reached consensus on code meanings and applications. The coders developed the code book and analytical framework by defining the codes (from open coding) and grouping them into working categories. The coding dictionary and analytical framework were supplemented with and guided by codes reflecting MCP concepts (and definitions), including attitudinal, historical, creative, and experiential sources of meaning. (5) The coders applied the analytical framework; the three coders coded all the responses and met to discuss points of divergence and convergence until the group reached consensus. Intercoder reliability was conducted through team-based consensus building. (6) Using a spreadsheet to generate a matrix, the qualitative data (quotations) were “charted” into a matrix of the analytical framework. (7) The data were discussed and interpreted for the generation of this research article. All the investigators had expertise in qualitative analysis, and the first author moderated these discussions.

## Results

The sample (see Table 1) included 142 Latinos with advanced cancer (67.6% stage IV and 32.4% stage III), and the most frequent diagnoses were breast (36.6%) and gastrointestinal (16.2%) cancers. The mean age was 57 years. Most patients were female (67.8%), slightly more than half were married or partnered (53.2%), almost one third had less than a high school education (31.9%), and 41.9% had at least some college education. Slightly more than half of the sample reported being unemployed or on disability (52.1%). All spoke fluent Spanish, and most reported Spanish language preference (85.3%). The participants were born predominantly in the Dominican Republic (31.7%), Puerto Rico (28.2%), and the continental USA (10.6%).

**Table 1.** Characteristics of Latinos with advanced cancer who participated in the study

	Participants, No. (%)^a^	
Characteristic	Total (*N* = 142)^b^	Qualitative cohort (*n* = 24)
Age, y	–	–
Mean (SD)	56.6 (12.3)	54.17 (13.7)
Sex	–	–
Male	46 (32.2)	16 (66.7)
Female	97 (67.8)	8 (33.3)
Marital status	–	–
Married or partnered	75 (53.2)	20 (83.3)
Single	23 (16.3)	3 (12.5)
Separated	11 (7.8)	0 (0.0)
Divorced	25 (17.7)	1 (4.2)
Widowed	7 (5.0)	0 (0.0)
Education level	–	–
Less than high school	45 (31.9)	6 (25.0)
12th grade/high school graduate	35 (24.8)	6 (25.0)
Some college or associate’s degree	33 (23.4)	8 (33.3)
College graduate	18 (12.8)	2 (8.3)
Post-college/graduate school	8 (5.7)	2 (8.3)
Employment status	–	–
Employed	32 (22.5)	12 (50.0)
Retired	34 (23.9)	6 (25.0)
Unemployed or disabled	74 (52.1)	6 (25.0)
Race	–	–
White or Caucasian	39 (28.1)	11 (45.8)
Black	11 (7.9)	1 (4.2)
Indigenous	2 (1.4)	0 (0.0)
Other	86 (61.8)	12 (50.0)
Preferred language	–	–
English	122 (85.3)	–
Spanish	16 (11.2)	21 (87.5)
Both	5 (3.5)	3 (12.5)
Birthplace	–	–
United States	15 (10.6)	2 (8.3)
Puerto Rico	40 (28.2)	10 (41.7)
Dominican republic	45 (31.7)	3 (12.5)
Mexico	8 (5.6)	2 (8.3)
Other countries in Latin America	34 (24.0)	7 (29.2)
Diagnoses	–	–
Breast	52 (36.6)	6 (25.0)
Prostate	12 (8.5)	5 (20.8)
Gastrointestinal	23 (16.2)	3 (12.5)
Lung	10 (7.0)	0 (0.0)
Other	45 (31.7)	10 (41.7)
Cancer stage	–	–
III	46 (32.4)	11 (45.8)
IV	96 (67.6)	13 (54.2)

a Values are no. (%), unless otherwise noted.

b Frequencies may not be based on the total sample, due to missing data, and percentages may not equal 100%, due to rounding.

### Association of spiritual well-being with psychological adjustment

Spiritual well-being as a whole and both factors (faith and peace/meaning) were associated with lower anxiety (*r* = −0.53, *p* < .001; *r* = −0.27, *p* = .001; *r* = −0.58, *p* < .001, respectively), depression (*r* = −0.62, *p* < .001; *r* = −0.37, *p* < .001; *r* = −0.64, *p* < .001, respectively), combined anxiety and depressed mood (*r* = −0.63, *p* < .001; *r* = −0.36, *p* < .001; *r* = −0.67, *p* < .001, respectively), hopelessness symptoms (*r* = −0.60, *p* < .001; *r* = −0.29, *p* = .001; *r* = −0.66, *p* < .001, respectively) and with higher quality of life (*r* = 0.62, *p* < .001; *r* = 0.33, *p* < .001; *r* = 0.68, *p* < .001, respectively). The meaning/peace dimension showed stronger negative associations with emotional suffering than the faith dimension. In multivariate regression models controlling for age, education, marital status, and time since diagnosis, spiritual well-being significantly predicted higher quality of life (*β* = .81, *p* < .001) and lower depression level scores (*β* = −.26, *p* < .001).

Further, in models including the meaning/peace and faith subscales (Table 2), the meaning/peace factor showed a stronger relationship with mood, anxiety, depression, hopelessness symptoms, and quality of life level scores (*β* = −.85, *p* < .001; *β* = −.41, *p* < .001; *β* = −.44, *p* < .001; *β* = −.35, *p* < .001; *β* = 2.20, *p* < .001; respectively). Faith had no relationship with any of the symptoms (mood, anxiety, depression, and hopelessness) or quality of life when controlling for the meaning/peace factor.

**Table 2.** Multivariate regression analyses predicting quality of life and depression

	Quality of life				
	*F*	*R*^2^	Beta	SE	*p*
Mood^a^	11.50	0.43	–	–	.000
Faith	–	–	−.025	.177	.890
Meaning/Peace	–	–	−.848	.107	.000
Anxiety^a^	7.94	0.34	–	–	.000
Faith	–	–	.016	.103	.873
Meaning/Peace	–	–	−.409	.062	.000
Depression^a^	10.49	.41	–	–	.000
Faith	–	–	−.039	.102	.701
Meaning/Peace	–	–	−.438	.061	.000
Hopelessness^a^	13.93	.48	–	–	.000
Faith	–	–	.002	.077	.984
Meaning/Peace	–	–	−.350	.046	.000
Quality of life^a^	13.72	.46	–	–	.000
Faith	–	–	.144	.429	.738
Meaning/Peace	–	–	2.204	.267	.000

a Controlling for age, education, marital status, and birth country.

### Acceptability of existential themes: Quantitative analysis

Most patients reported the following strategies as “quite a bit” to “extremely” important for coping with the cancer diagnosis (see Table 3): Maintaining hope (87.3%); Being responsible for myself after my cancer diagnosis (83.8%); Understanding what the purpose is of my life after being diagnosed with cancer (80.3%); Making-meaning in my life or thinking about my purpose in life (79.6%); and Making sense of the cancer experience (78.2%). Additionally, most patients found the following MCP sources of meaning items as acceptable: The love that I have for my loved ones has helped me to cope with my cancer diagnosis (82.4%); Maintaining a good sense of humor has helped me to cope with my cancer diagnosis (78.9%); The love that I have for life has helped me to cope with my cancer diagnosis (76.8%); and Finding beauty in music, nature and other life experiences has helped me to cope with my cancer diagnosis (76.8%). Finally, the most frequently endorsed important themes of counseling were: Coping with cancer (95.1%), changes in life after cancer (95.1%), finding purpose in life (89.4%), family issues (82.4%), spiritual issues (74.6%), and religious issues (71.8%).

**Table 3.** Importance and acceptability of existential themes (*N* = 142)

Themes	No.	(%)
**Quite a bit to extremely important strategies for coping with patient’s cancer diagnosis**		
Maintaining hope	124	(87.3)
Being responsible for myself after my cancer diagnosis.	119	(83.8)
Understanding what the purpose is of my life after being diagnosed with cancer	114	(80.3)
Making meaning in my life or thinking about my purpose in life	113	(79.6)
Making sense of the cancer experience	111	(78.2)
Reflecting or thinking about how I have changed after my cancer diagnosis	109	(76.8)
Changing or adjusting my attitude when circumstances are beyond my control.	103	(72.5)
Taking care of others after my cancer diagnosis.	103	(72.5)
Reflecting on my heritage or thinking about what I have contributed with my life	92	(64.8)
**Sources of meaning**		
I think that the love that I have for my loved ones has helped me to cope with my cancer diagnosis	117	(82.4)
I think that maintaining a good sense of humor has helped me to cope with my cancer diagnosis	112	(78.9)
I think that the love that I have for life has helped me to cope with my cancer diagnosis	109	(76.8)
I think that finding beauty in music, nature and other life experiences has helped me to cope with my cancer diagnosis	109	(76.8)
**I would like to receive support to:**	–	–
Find and maintain hope to cope with my cancer diagnosis	99	(69.7)
Make sense or find meaning in life after my cancer diagnosis.	84	(59.2)
To do a life review, thinking about my heritage and my life contributions	65	(45.8)
**Important or very important themes for therapy/counseling**	–	–
Coping with cancer	135	(95.1)
Changes in life after cancer	135	(95.1)
Finding purpose in life	127	(89.4)
Family issues	117	(82.4)
Spiritual issues	106	(74.6)
Religious issues	102	(71.8)

### Meaning-making and sources of meaning themes: Qualitative analyses

Themes about meaning-making and sources of meaning as used in MCP (see Table 4) were qualitatively explored. Recurrent themes included meaning-making coping, attitudinal sources of meaning, social/family sources of meaning, spiritual and religious sources of meaning, historical sources of meaning-legacy, creative sources of meaning, and experiential sources of meaning.

**Table 4.** Qualitative results

Categories	Codes	Endorsement number	Exemplary quotes
**Sources of meaning: Attitude**	Choose a positive attitude	12	… Positive, positive. While you’re more positive, and say “I’m going to overcome, I’m going to overcome,” I think that you give it less mind … .
			It is logical that it can affect me. But if you don’t instantly reconsider and if you become negative, the illness will knock you down more. So I try to think positively so that the illness doesn’t take me away.
	Self-love	2	The sense that I am finding towards myself in my way of being – it was a bit, like a bit negative, and now I feel a bit more positive.
			First of all, you have to love God and yourself.
	Enjoying life/passion	8	Because there are so many projects at the same time that everything passes by … That doesn’t mean I haven’t been planning for the future. But I’m enjoying my present … .
			The purpose of my life is… trying to move on … with my family. To do the things … go out, walk, and have fun.
	Maintain a fighting spirit/fight	10	I have to continue moving forward and continue with … my life …
			I have enough in life to want to continue the battle, no? To see in my children that they fulfill and help them achieve their goals. My siblings, my friends, my projects …
	Surviving	8	My purpose is to continue living. To continue, to fight against cancer … . That is the purpose. My goal is also that maybe I’ll be cured and continue the activities much better.
**Sources of meaning: social and family**	Family	18	To love the family, my children, my grandchildren, my siblings, right? And all the people, well those who love me, although sometimes we feel rejected too …
	Happiness of children/family	6	Oh … when I see them happy! Me Seeing my children happy is everything to me.
			When my grandchildren are near me, right? It changes when they send me a photo of them, when my daughter also sometimes sends me photos …
	Helping others	6	It’s my new purpose that I have in helping a 100% this man (my doctor) so that he has the best possible, so that even if I die, well, that, as he says, it can help the next generations. You know, to my nephew or whoever it is, so they don’t have that problem. Just like it happened with polio and as it happened with all those other things, right.
			My purpose is … to fight for my husband. Help those people who need me, who are sick … to offer them my hands.
**Spiritual and religious sources of meaning**	Religiosity	5	Now, my only hope is in God, and I am Catholic. I know there is no cure, but thinking about God calms me down a lot. Before, I was a Catholic by name, but now I go to prayer groups, talks, and it helps me a lot.
	Spirituality	13	Spirituality. Being calm with myself, being well, not having fear. I’m not afraid … . Thanks for what I had to live through and to where I have to go. Nothing else.
			Yes, I do consider myself a spiritual person and that has helped me. Because I feel that I have … the meaning I think is … to be the best person that one can be.
	Spiritual growth	6	I thank them for falling ill, because I have learned to grow more, morally and spiritually.
	Trust in God	2	Him, all because if I didn’t trust in God, I believe that I wouldn’t even have the strength to be here right now or anything else.
**Historical sources of meaning-legacy**	Legacy to the children/ education	1	A legacy that I have given them? That they study. That they become professionals … and that I feel calmer.
	Legacy: Family union	1	… It’s just that many times, when the tree trunk dies, I already know, branches separate a bit, right? But then I have wanted … them to, I want them to live together forever, even though one of them is already married, but he … may they always stick together, because they don’t have family here. And they always stick together.
	Legacy: Being remembered positively	1	Be remembered in a positive way. I know there are moments in my life, things, everyone makes mistakes. There are things I wish I could erase. But, they can’t be.
**Creative sources of meaning**	Work	6	Don’t just keep a single income, or that a person has an investor mentality because they have multiple incomes, one has to have more income than expenses, and you know everything is like … looking for a quality life.
	Having goals and projects	3	There are many goals to achieve and they are not short-term. They are goals that take time, because I haven’t achieved them yet, since I know I’m still involved with them.
	Music	3	Well, my son. My son just turned one. I’m a musician. I honestly feel that music, it, it is one of the best remedies, I think. Because, somehow, I don’t know, it always keeps my mind busy …
**Experiential sources: Love, beauty, nature, and humor**	Sources of love: Family	4	The sources of love in my life, I mentioned them before … my husband, my children, and God, first and foremost God.
			No, no. It did change the meaning with my wife … and I think that this has given us the opportunity to have a much closer relationship. If it was close before, it’s much more now.
	Sources of beauty: Love	1	I’ve always been very sensitive to this, this way love, beauty, and everything one wants, one can always find beauty one way or another … but, I have much love for people that are around me.
	Sources of beauty: Nature	3	Yes … in nature, in the animals, in children.
	Sources of beauty: Music	3	Music and composing. But look, my hands are numb because of chemo … I know I can and will get better. And if I don’t, I’ll find a way. I’ll wear gloves, I’ll find a way …
	Sources of beauty: Children	1	Seeing a child, a baby, seeing kids having fun …
	Sources of beauty: Animals	1	Yes … in nature, in the animals …
	Sources of happiness: Children’s and family’s happiness	1	Oh … when I see them happy! Seeing my children happy means everything to me. There’s nothing greater than seeing them … happy.
	Sources of happiness: Enjoying life	7	Enjoy, go out, walk, and have fun. Maybe make plans for my vacation trips and be closer to my children too, who are so distant, going to visit them.

When prompted about the main ways patients find meaning and purpose in their lives, the most shared themes included self-love, love for others, spirituality, experiential sources of meaning in life, and attitude/hope.

**Attitudinal sources of meaning.** When patients were asked about the ways they find meaning and purpose in choosing their attitude to face life situations, the main themes that emerged were positive attitude, self-love, love for life, enjoy life (passion about life), fighting spirit, surviving, finding purpose in the situation, and active learning. An exemplary quote of choosing a positive attitude was, “It is logical that it can affect me. But if you don’t instantly reconsider and if you become negative, the illness will knock you down more. So, I try to think positively so that the illness doesn’t take me away.”

**Social/family sources of meaning.** Patients often shared that they find meaning through social and family connections, including love for the family, happiness of children and family, joy for having loved ones, helping others, and being an example for others. A quote illustrating family connection is, “To love the family, my children, my grandchildren, my siblings, right? and all the people, well, that love me, although sometimes we feel rejected too… .”

**Spiritual and religious sources of meaning.** Spirituality, religiosity, spiritual growth, and trusting in God were cited as sources of meaning for many patients. A quote that illustrates spirituality is “Spirituality being calm with myself, being well, not being afraid. I’m not afraid. Thanks for what I had to live through and for where I have to go. Nothing else… .”

**Other sources of meaning.** Some of the creative sources of meaning cited by patients were work, having goals and projects, and music. Some of the experiential sources of meaning included loving the family and finding beauty in love, nature, music, children, or animals. Sources of joy and humor included children and family happiness, and enjoying life. A few patients shared their legacy (historical sources of meaning), which included supporting the academic goals of their children, family cohesion (union), and being remembered in a positive light. The meaning-making ways of coping and/or sources of meaning cited more frequently (by eight or more patients) were choosing a positive attitude (*n* = 12), enjoying life (*n* = 8), fighting spirit (*n* = 10), surviving (*n* = 8), family (*n* = 18), and spirituality (*n* = 13). Furthermore, family happiness, helping others, spiritual growth, work, and religiosity were each cited by five patients.

## Discussion

This study examined associations between adjustment and spiritual well-being, meaning/peace, and faith in Latinos with advanced cancer. It explored the importance of existential themes and coping, and the narratives of meaning-making and sources of meaning. Spirituality is a helpful coping resource for many Latinos facing an advanced diagnosis (Breitbart 2002; Lin and Bauer-Wu 2003; Troncoso et al. 2019). In this study, spiritual well-being and its two dimensions, meaning/peace and faith (related to spiritual beliefs), were associated with lower mood symptoms (depression, anxiety, and hopelessness) and better quality of life in Latinos with advanced cancer. However, only meaning/peace was associated with a significantly better improvement in quality of life and reduction of mood symptoms when controlling for the faith factor. Previous literature has shown the importance of faith and religiosity in the Latino community (Culver et al. 2002, 2004; Garduño-Ortega et al. 2021; Hunter-Hernandez et al. 2015; Moadel et al. 1999; Samuel et al. 2020), but our findings highlight that meaning/peace is more critical for emotional adjustment and overall well-being. Our findings are also consistent with results from a previous study involving Latina breast cancer survivors that found a stronger association between peace/meaning with depression and quality of life than faith (Garduño-Ortega et al. 2021).

Further, upon exploration of the acceptability of existential themes and existential coping in this sample of people with advanced cancer, it was revealed that maintaining hope, understanding purpose in life after the diagnosis, and making-meaning in life were themes endorsed by more than 80% of the participants as important. Most (89%) also stated that finding purpose in life was an important goal of counseling. These findings support the importance of spiritual coping in Latinos with advanced cancer, with a focus on existential coping, not only religious coping. Further, in our inquiry, a higher proportion endorsed addressing meaning or existential themes as a therapeutic goal over religious themes.

Lastly, the themes that were more frequently discussed in the qualitative interviews that explored meaning-making coping, and sources of meaning were attitudinal, social/family and spiritual sources of meaning. Considering that spirituality is a core cultural value for a large part of the Latino population (Janz et al. 2009), as it was for those in the current study, it is imperative to address and strongly encourage finding and maintaining meaning and peace (sense of meaning and purpose in life, and peace) while facing advanced cancer and end of life in Latino cancer patients. The roles of meaning and meaning-making have been extensively explored with non-Hispanic populations. Patients who report spiritual well-being (hope and meaning in life) are better equipped to cope with the process of terminal cancer and discovering meaning in the experience (Lin and Bauer-Wu 2003).

Further, finding meaning and purpose were often experienced through social and family connections, including love for the family, happiness of children and family, joy for having loved ones, helping others, and being an example for others. This finding is consistent with numerous studies showing that Latinos who feel supported by their family and friends adjust better to the diagnosis (Galván et al. 2009; Ochoa et al. 2018).

Our findings demonstrate the importance of implementing a meaning/peace approach (sense of purpose and meaning in life, and peace) with Latinos with advanced cancer. Nelson et al. (2002) reported similar findings with predominantly non-Latino White patients with terminal cancer and AIDS, and Garduño-Ortega et al. (2021) with a sample of Latina breast cancer survivors. Both studies found that meaning/peace was a crucial factor, more critical than faith. Our study also revealed that the major ways that patients found meaning and purpose in life were through their attitude, love for others (social/family connections), spirituality, and engagement with life.

This study suggests that implementing a meaning/peace approach may positively impact the adjustment of Latino patients with cancer. Psychotherapy interventions such as MCP (Breitbart et al. 2018, 2012) and other existential-based therapies, such as Dignity Therapy (Chochinov et al. 2005), supportive-expressive therapy (Kissane and Li 2008), and family grief therapy (Kissane et al. 2006), have been developed to address meaning-making needs and to encourage a sense of dignity and purpose in life at the end of life. Those interventions aimed at improving psychological adjustment have proven effective in predominantly non-Latino White people with cancer. Studies to adapt and implement MCP for Latino people with cancer (Costas-Muniz et al. 2020, 2023) and Latino caregivers (Torres-Blasco et al. 2022) are underway. Meaning-making needs should be addressed in clinical settings. Health professionals can play an essential part by identifying patients’ needs and referring patients and families to programs for help.

There were some limitations to this study. First, the patients were recruited from New York City and Puerto Rico, with a sample of people predominantly born in the Caribbean. The findings may not generalize to Latinos from other geographic locations in the USA and Latin America. Future studies should include patient samples recruited in different geographical locations to enhance cultural representation and fit of the intervention. Second, only patients with stage III and stage IV cancer were invited to participate. The cancer experience of patients at different disease stages with different prognoses could vary significantly. The findings might not be applicable to patients with early-stage disease. In future studies, analyses stratified by stage and prognosis status are warranted.

## Editorial support

The authors thank Sonya J. Smyk, Memorial Sloan Kettering Cancer Center, for editorial support. She was not compensated beyond her regular salary.
